# Closing the gap in the clinical adoption of computational pathology: a standardized, open-source framework to integrate deep-learning models into the laboratory information system

**DOI:** 10.1186/s13073-025-01484-y

**Published:** 2025-05-26

**Authors:** Miriam Angeloni, Davide Rizzi, Simon Schoen, Alessandro Caputo, Francesco Merolla, Arndt Hartmann, Fulvia Ferrazzi, Filippo Fraggetta

**Affiliations:** 1 Institute of Pathology, Friedrich-Alexander-Universität Erlangen-Nürnberg (FAU), Erlangen, Germany; 2 Comprehensive Cancer Center Erlangen-EMN (CCC ER-EMN), Erlangen, Germany; 3 Bavarian Cancer Research Center (BZKF), Erlangen, Germany; 4 TESI Group/GPI, Milan, Italy; 5 Department of Pathology, University Hospital of Salerno, Salerno, Italy; 6 Department of Medicine and Surgery, University of Salerno, Salerno, Italy; 7 Department of Medicine and Health Sciences “Vincenzo Tiberio”, University of Molise, Campobasso, Italy; 8 Department of Nephropathology, Institute of Pathology, Friedrich-Alexander-Universität Erlangen-Nürnberg (FAU), Krankenhausstr. 8-10, Erlangen, 91054 Germany; 9 Unit of Pathology, Gravina Hospital, Via Portosalvo 1, Caltagirone, 95041 Italy

**Keywords:** Anatomic pathology laboratory information system, Computational pathology, Deep-learning models, Digital pathology, HL7, Integration

## Abstract

**Background:**

Digital pathology (DP) has revolutionized cancer diagnostics and enabled the development of deep-learning (DL) models aimed at supporting pathologists in their daily work and improving patient care. However, the clinical adoption of such models remains challenging. Here, we describe a proof-of-concept framework that, leveraging Health Level 7 (HL7) standard and open-source DP resources, allows a seamless integration of both publicly available and custom developed DL models in the clinical workflow.

**Methods:**

Development and testing of the framework were carried out in a fully digitized Italian pathology department. A Python-based server-client architecture was implemented to interconnect through HL7 messaging the anatomic pathology laboratory information system (AP-LIS) with an external artificial intelligence-based decision support system (AI-DSS) containing 16 pre-trained DL models. Open-source toolboxes for DL model deployment were used to run DL model inference, and QuPath was used to provide an intuitive visualization of model predictions as colored heatmaps.

**Results:**

A default deployment mode runs continuously in the background as each new slide is digitized, choosing the correct DL model(s) on the basis of the tissue type and staining. In addition, pathologists can initiate the analysis on-demand by selecting a specific DL model from the virtual slide tray. In both cases, the AP-LIS transmits an HL7 message to the AI-DSS, which processes the message, runs DL model inference, and creates the appropriate visualization style for the employed classification model. The AI-DSS transmits model inference results to the AP-LIS, where pathologists can visualize the output in QuPath and/or directly as slide description in the virtual slide tray.

**Conclusions:**

Taken together, the developed integration framework through the use of the HL7 standard and freely available DP resources offers a standardized, portable, and open-source solution that lays the groundwork for the future widespread adoption of DL models in pathology diagnostics.

**Supplementary Information:**

The online version contains supplementary material available at 10.1186/s13073-025-01484-y.

## Background

The advent of digital pathology (DP), with the digitization of histopathology glass specimens into high-resolution whole-slide images (WSIs), has revolutionized cancer diagnostics and research [1]. The advantages offered by WSIs are manifold, ranging from an improved laboratory workflow, to remote consultation, rapid second opinions, teaching, and training [2]. Importantly, the digitization of glass slides enabled the development and application of numerous artificial intelligence (AI)-based tools, initiating a paradigm shift towards the adoption of computer-assisted diagnostic systems [3]. In precision oncology, AI tools have proven capable of tumor detection, subtyping, classification, as well as prediction of genetic alterations and cancer prognosis [4].

Over the past 10 years, the number of published AI-based computational pathology (CPath) tools has increased more than 100-fold [4]. Nevertheless, the vast majority of them are rarely used in clinical practice [5]. Several reasons have hampered the adoption of both commercial and non-commercial tools in routine diagnostics. Firstly, their clinical use would require a fully digital workflow. However, due to cultural and technical challenges [6, 7], few anatomic pathology departments are fully digital. The lack of prospective clinical validation as well as the regulatory approval required for the clinical use of AI-based diagnostic assays represent further limitations [4, 6]. In addition, using AI in routine diagnostics is typically not yet reimbursed [4, 8] and thus constitutes a financial burden for healthcare institutions. Also, the establishment of a protocol for integrating CPath solutions within the anatomic pathology laboratory information system (AP-LIS) is crucial [4]. Many commercial deep-learning (DL) models run on cloud servers, and often rely on proprietary viewers, whose integration within the existing AP-LIS is non-trivial. Most of the publicly available DL models, on the other hand, are not easily reusable [9], not even in fully digitized diagnostic workflows, nor accessible to pathologists without programming skills [10]. Furthermore, AI solutions often appear as black-box tools as the output consists only of class prediction probabilities. Studies tackling the effort of making AI tools usable in routine diagnostics exist [11–15], but a seamless integration into the AP-LIS represents a significant challenge still to be addressed.

Here, we developed the first standardized, open-source prototype framework for integrating both publicly available and custom developed DL models into the AP-LIS relying on the internationally recognized Health Level 7 (HL7) standard [16] and open-source DP software. To this aim, we leveraged the unique infrastructure offered by the Caltagirone pathology department [17], which shifted towards a fully digitized diagnostic workflow in 2019 by adopting a lean approach and implementing a fully tracked pathology system integrated within the AP-LIS. In our framework, HL7 is used to interconnect the DL models to the AP-LIS, and open-source DP software is employed both for running DL model deployment on routine slides and visualizing DL model inference results. Our results show how three key challenges limiting the clinical adoption of CPath solutions can be successfully addressed, namely (i) establishing an integration framework, (ii) including publicly available DL models, and (iii) implementing an intuitive visualization strategy for model inference results. This work is, to the best of our knowledge, the first proof-of-concept study aiming at bridging the gap in the clinical adoption of diagnostic and prognostic DL models, thereby marking an important step to move beyond academic research.

## Methods

### Caltagirone’s pathology department infrastructure

Development and testing of the integration framework was carried out in a fully digitized Italian pathology department at Gravina Hospital in Caltagirone, Italy [17]. Here, four different scanners are in use to digitize routine glass slides into WSIs: Panoramic P1000 (3DHistech, Budapest, Hungary) with a resolution of 0.24 microns per pixel (mpp), Panoramic P250 (3DHistech) with a resolution of 0.24 mpp, Aperio AT2 (Leica Biosystems Imaging, California, USA) with a resolution of 0.25 mpp, and Aperio GT 450 (Leica Biosystems Imaging) with a resolution of 0.26 mpp. Slides are stored in the respective scanner manufacturer’s proprietary image format (.mrxs and.svs) on a network-attached storage (NAS) system (Qnap NAS TVS-EC1280U-SAS-RP) via a 100Mbit/s network connection. The digital workflow uses the laboratory management application software Pathox (v13.32.0, Tesi Elettronica e Sistemi Informativi S.P.A., Milan, Italy), which enables WSIs to be opened directly from the virtual slide tray of the AP-LIS and visualized by default in the scanner-specific viewing software. In April 2023, QuPath [18] was additionally integrated within the AP-LIS.

### Whole-slide images acquisition and characteristics

Within our framework, a total of 11,805 hematoxylin and eosin (H&E) slides digitized at the Gravina Hospital pathology department as part of the routine diagnostic activity underwent deployment with one or more DL models in the period of time ranging from April 2024 to January 2025. These slides represented various specimen types, e.g., biopsy or surgical resection specimens, and belonged to 3157 patients, with a mean number of 3.5 slides (range, 1–57) per patient. Acquisition of WSIs for the development and testing of the integration framework occurred within the same routine clinical scenario used for diagnosis. No deviation from the standard of care occurred and no additional patient clinical information were collected.

### Computational hardware and software

All computational tasks, including the implementation of the Python-based server-client architecture and DL models deployment, were performed on a remote server located in the Gravina Hospital, equipped with two AMD EPYC 7313 processors and two AMD Radeon Instinct MI210 (64 GB RAM) graphics processing units (GPUs) and installed with Ubuntu’s 22.04.4 long-term support (LTS) operating system. The integration framework was developed in a dedicated conda environment with Python v3.10.14. To leverage AMD GPUs, the PyTorch DL framework was installed with Radeon Open Compute (ROCm) support (v5.7) together with the libraries torch v2.3.1 [19], and torchvision v0.18.1 [20]. For deployment of pre-trained DL models, one of the two GPUs was used.

Pathologists’ workstations to visualize the output of DL model inference results included computers with consumer-grade monitors, a QuPath installation and hardware specifications as previously described [17].

### Deployment framework for patch-level and slide-level pre-trained deep-learning models through WSInfer and WSInfer-MIL

Different types of DL models were integrated in the developed framework, including both strongly supervised learning frameworks (hereafter referred to as “patch-level” classification models) and weakly supervised [21] learning frameworks, such as attention-based multiple instance learning (MIL) [22] approaches (hereafter referred to as “slide-level” classification models).

Deployment of pre-trained patch-level classification models was performed relying on the WSInfer [10] command-line (CLI) tool v0.6.1. WSInfer was installed in the dedicated conda environment via pip after PyTorch installation, as recommended by the authors [23, 24]. The tool provides a fast, end-to-end workflow that (i) segments the tissue in the WSI, (ii) creates patches of the segmented tissue region, (iii) runs the pre-trained model on the extracted patches, and (iv) saves model results, with minimal, if any, user intervention. WSInfer allows the deployment of both built-in and customized DL models. Pre-trained weights for built-in models, with the associated configuration files, were retrieved through the Python package wsinfer-zoo v0.6.2 [25], which was automatically installed as dependency during the WSInfer pip installation. Deployment of WSInfer built-in models was performed by running the wsinfer run command with openslide v3.4.1 as backend for loading WSIs (--backend = openslide argument) and relying on the default settings for both batch size (--batch-size argument) and number of workers (--num-workers argument). For WSInfer built-in models, the wsinfer run command was launched using the following required arguments: a directory of WSIs (--wsi-dir argument), a directory where to store results (--results-dir argument), and the name of the DL model to deploy (--model argument). For inference with customized DL models, the model to deploy was instead specified by adding to the wsinfer run command the path to the local model configuration JSON file (--config argument), and the path to the pre-trained local model in TorchScript format (--model-path argument). The wsinfer run command creates, by default, a structured output folder storing, for each WSI: the tissue mask as JPEG file, an h5 file containing patch coordinates, a JSON file storing the metadata of the run, and a CSV file storing model inference results. This CSV file contains a row for each tile, with columns storing the x and y coordinates of the tile in base pixel units, its height and width, and its prediction score, one for each class. All the eight built-in models provided by the WSInfer CLI tool were included in the integration framework.

Deployment of pre-trained MIL models for slide-level classification tasks was performed relying on the WSInfer-MIL CLI tool v0.1.0 [26], which was installed in the dedicated conda environment via pip following authors’ instructions. Analogously to the WSInfer patch-level counterpart, WSInfer-MIL provides a streamlined end-to-end workflow that operates at whole-slide level and goes from tissue detection to model prediction. Also, it allows deploying both built-in and customized DL models. Inference with built-in models was performed running the wsinfer-mil run command with the following required arguments: the Hugging Face Hub repository ID of the model (--hf-repo-id argument), and the path to the WSI (--wsi-path argument). To run model inference on mrxs files, the modules wsi_utils.py, inference.py, and data.py of the wsinfer-mil package were modified to replace tiffslide with openslide for WSI opening and reading. Furthermore, differently from the WSInfer CLI-tool, WSInfer-MIL does not save slide-level predictions in a CSV file, but prints them directly in the command prompt. Thus, to streamline the post-processing of DL model inference results, and allow their visualization, the output_container.py and the inference.py modules were modified to save both slide-level and patch-level predictions in a CSV file. In the CSV file with slide-level predictions, the number of rows was taken equal to the number of classes, with the first column storing the class name, and the second column containing the prediction probability for each class. In the CSV file with tile-level predictions, the number of rows was instead taken equal to the number of tiles, and six columns were created to store, for each tile, its x and y coordinates in base pixel units, its height and width, its attention score, and its percentile-ranked attention score. Percentile ranked attention scores were calculated, starting from the un-normalized attention scores, using the Pandas rank() method and setting the argument “pct” as true. Three WSInfer-MIL built-in models were included in the integration framework: pancancer-tp53-mut.tcga [27], gbmlgg-survival-porpoise.tcga [28, 29], and kirp-survival-porpoise.tcga [28, 30]. For survival models, the formula to calculate the risk score based on model output logits, as well as the median risk score to split patients into low risk and high risk, were retrieved from the corresponding model’s huggingface.com page.

### Deployment framework for slide-level pre-trained deep-learning models through marugoto

For slide-level classification tasks, our integration framework includes also marugoto v0.8.0 [31] as additional toolbox. In particular, the integrated pre-trained slide-level classification models for the assessment of *BRAF* mutational status and microsatellite instability (MSI) relied on marugoto for deployment. For both classification tasks, the file export-0.pkl contained in Wang + attMIL/BRAF and Wang + attMIL/isMSIH subfolders was chosen for inference on new WSIs [32, 33].

marugoto was installed in the same conda environment hosting WSInfer and WSInfer-MIL CLI tools by first cloning the GitHub repository [31] and then running the command “pip install” from the marugoto subfolder, as specified in the documentation [34]. Similarly to WSInfer-MIL, marugoto provides a framework for weakly supervised classification problems that goes from feature extraction to model deployment on pre-extracted features. However, differently from WSInfer and WSInfer-MIL, the framework does not include tissue segmentation and patches generation. Instead, it assumes that features extraction is applied to previously extracted patches saved in JPEG format. To make tissue segmentation and patches generation as efficient as possible, and to avoid storing patches as JPEG files, the module xiyue_wang for feature extraction was customized to take as input the h5 file in output from the WSInfer CLI tool containing patch coordinates. Thus, all WSIs analyzed with marugoto first underwent tissue segmentation and patches generation through the WSInfer command wsinfer patch, and then the output h5 file with patches coordinates was used as input for running the marugoto.extract.xiyue_wang features extraction module. To generate patches coordinates, the wsinfer patch command was run using the following required arguments: a directory of WSIs (--wsi-dir argument), a directory where to store results (--results-dir argument), the patch size in pixel (--patch-size-px argument), and the physical spacing of the patches in mpp (--patch-spacing-um-px argument). A patch size of 224 pixel and a patch spacing value of 1.14 mpp were used for patches generation [33]. Finally, model deployment on the pre-extracted features was performed relying on the marugoto.mil deploy module. The module was run using the target label “BRAF” (--target_label argument) and the categorical labels [“MUT”,“WT”] (--cat_labels argument) for the assessment of *BRAF* mutational status, and using the target label “isMSIH” and the categorical labels [“MSIH”,“nonMSIH”] for the assessment of MSI status. As additional input, each time one of these two models is deployed on a new slide, two CSV files are created. The first CSV file (--clini_table argument) contains one column called “PATIENT,” storing patient identifier, and one column named as the target label (i.e., BRAF or isMSIH) containing one of the two associated categorical labels. The second CSV file (--slide-csv argument) contains one column called “PATIENT,” storing the same patient identifier found in the clinical table, and one column called “FILENAME” with the filename, without extension, of the h5 file storing feature vectors. Similarly to WSInfer-MIL, to visualize the results of DL model inference, the patch-level predictions were saved in CSV format. To this aim, the get_dict_maptype_to_coords_scores function from the module marugoto.visualizations.mil_heatmaps was modified to return the un-normalized attention scores as additional output. For each analyzed slide, tile coordinates, un-normalized attention scores, and normalized attention scores were obtained by running the customized get_dict_maptype_to_coords_scores function with MayType “ATTENTION.” Percentile ranked attention scores were then calculated, starting from the un-normalized attention scores, using the Pandas rank() method and setting the argument “pct” as true. Finally, the CSV file containing the patch-level predictions was created with a number of rows equal to the number of tiles, and seven columns that, for each tile, store: x and y coordinates in base pixel units, height, width, attention score, normalized attention score, and percentile-ranked attention score.

### Visualization of deep-learning model inference results in QuPath

DL model inference results were visually represented as colored heatmaps relying on the open-source bio-image analysis software QuPath [18] v0.4.3 and on the Python package paquo v0.8.0 [35], which provides a pythonic interface to create and work with QuPath projects.

For each analyzed WSI, a new QuPath project (.qpproj file) was instantiated using the QuPathProject class from the paquo.projects module, and populated with the WSI through the QuPathProject.add_image() method. Tile detections were added to the project hierarchy using the QuPathPathObjectHierarchy.add_tile() method from the paquo.hierarchy module. Each tile was drawn as a polygon using the Polygon.from_bounds() method of the Python package shapely v2.0.4. For 3DHistech slides (.mrxs), to correctly build tile detection objects in QuPath, the openslide patch coordinates generated by WSInfer and WSInfer-MIL CLI tools needed to be offset. To this aim, x and y offsets were automatically extracted for each WSI through the openslide.bounds-x and openslide.bounds-y properties, and subtracted from the original patch coordinates. The offset coordinates were then used as input for the Polygon.from_bounds() class method. Three different visualization styles were implemented, namely: measurement maps, color maps, and density maps.

In measurement maps, each tile was added to the project hierarchy using: (i) the polygon defined with Polygon.from_bounds() as value for the parameter “roi,” (ii) a pre-defined label (e.g., the worst label from the clinical point of view) among DL model categories as value for the parameter “path_class,” and (iii) the corresponding prediction score as value for the parameter “measurements.”

In color maps, a number of new classes equal to the number of categories of the DL model, each associated with a color pair, were defined within the QuPath project. The new classes were instantiated using the QuPathPathClass class from the paquo.projects module and assigned to the project through the QuPathProject.path_class property. Tiles were added to the project hierarchy analogously to measurement maps, respectively using as values for “path_class” and “measurements” the predicted label and the associated prediction score, determined by taking the argmax of the predictions across all classes.

For density maps, in addition to tile detection objects, a number of detection points proportional to the percentile-ranked attention score of each tile was created in correspondence of the tile’s center point. Notably, nine detections were drawn for scores > = 0.9, eight detections for scores between 0.9 and 0.8, and so forth until reaching a minimum of one detection point for percentile-ranked attention scores lower than 0.2. Detection points were created relying on the Point and MultiPoint functions of the shapely package and added to the project hierarchy through the QuPathPathObjectHierarchy.add_detection() method from the paquo.hierarchy module. Furthermore, a brief description of DL model inference results, including the slide-level predicted label/risk class and the associated predicted score/risk score, was added to the QuPah project as free text through the QuPathProjectImageEntry.description property of the paquo.hierarchy module.

### Interfacing between the AP-LIS and the AI-DSS through HL7 messaging

**Fig. 1:**
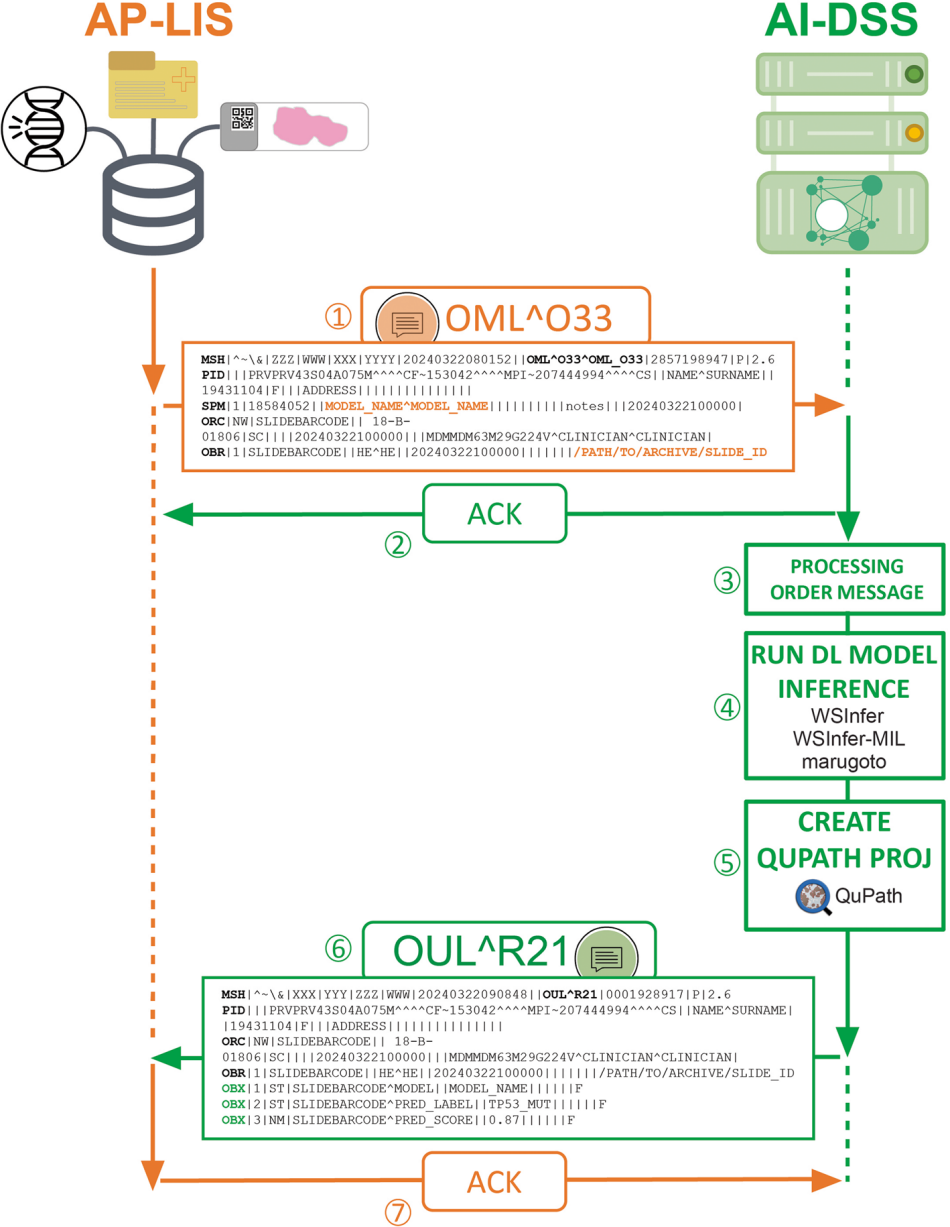
Schematization of the integration framework. The anatomic pathology laboratory information system (AP-LIS) and the external artificial intelligence (AI)-based decision support system (AI-DSS) containing a set of pre-trained deep-learning (DL) models communicate with each other via Health Level 7 (HL7) messaging. The communication occurs through the following sequence of events: (1) a laboratory order message (OML^O33) is sent from the AP-LIS to the AI-DSS; (2) an acknowledgment (ACK) message is sent from the AI-DSS to the AP-LIS upon reception of the OML^O33 message; (3) the AI-DSS processes the OML^O33 message retrieving information on the DL model name and the path to the whole-slide image (WSI) to analyze (bold orange); (4) DL model inference is run; (5) a QuPath project is built to visualize DL models results; (6) a laboratory observation message (OUL^R21) is sent from the AI-DSS to the AP-LIS. Here, DL model results are transmitted to the AP-LIS as OBX segments (bold green); (7) an ACK message is sent from the AP-LIS to the AI-DSS upon reception of the OUL^R21 message

Hereafter we will refer to the set of DL models interconnected to the AP-LIS as external AI-based decision support system (AI-DSS). Interfacing between the AP-LIS and the AI-DSS relied on the internationally recognized HL7 [16] version 2 protocol (v2.6) messaging (Fig. 1). The communication flow between the AP-LIS and the AI-DSS, as well as the adopted messaging, followed the HL7 standard Pathox_rev1_5 specifications. In particular, the interaction between the AP-LIS and the AI-DSS initiates in conjunction with an HL7 Laboratory Order Messages (OML^O33) request transmitted from the AP-LIS to the AI-DSS. Conversely, DL model inference results generated by the AI-DSS are transmitted to the AP-LIS via HL7 Unsolicited Laboratory Observation Messages (OUL^R21). In the implemented framework, each OML^O33 message corresponds to the analysis request of a single WSI, even when multiple WSIs are available for the same patient. This ensures a single “specimen” (SPM) segment and a single “common order”/“observation request” (ORC/OBR) segment pair. Two types of events can trigger an OML^O33 message: either the digitization of a new routine slide (“default” mode), or a request placed by pathologists through the virtual slide tray of the AP-LIS (“on-demand” mode). In the default mode, once a glass slide is digitized, the AP-LIS automatically receives the digitized slide together with all the associated information, including the tissue type (e.g., colon) and the staining (e.g., H&E). Then, on the basis of a pre-defined curated correspondence table between tissue type, staining, and DL models, as many OML^O33 messages as the number of available DL models for the given combination of tissue type and staining will be triggered. The correspondence table is updated as soon as new DL models are added to the AI-DSS. In the on-demand mode, the user chooses the model to deploy from a drop-down menu in the AP-LIS. For both deployment modes information about the DL model to employ and the location of the WSI to analyze are respectively stored in the fields SPM.4.1^SPM.4.2 and OBR.13 of the OML^O33 message (Additional file 1: Fig. S1). Of note, for slides digitized with 3DHistech scanners the location of the WSI is the folder containing the DAT files and the Slidedat.ini file, whereas for Leica scanners the location of the WSI is the location of the SVS file. All incoming OML^O33 messages are placed in a queue and, before any further processing, the AI-DSS transmits an acknowledgment (ACK) message to the AP-LIS. After the ACK message, the processing of the analysis requests follows the first in first out (FIFO) principle. For each message of the queue, first DL model’s name and WSI location are retrieved, then inference is run at whole-slide level with one of the available toolboxes, and ultimately a QuPath project is generated to display DL model inference results as a colored heatmap.

Basing on the type of pre-trained DL model used for deployment, the number of “observation/result” (OBX) segments of the output OUL^R21 message may vary (Additional file 1: Fig. S2). The first OBX segment always contains a string indicating the name of the model. In addition, for all WSIs where inference is performed with WSInfer, tissue mask (JPEG file), tile-level predictions (CSV file), and metadata about the run (JSON file) are added as distinct OBX segments encoded in Base64 (Additional file 1: Fig. S2A). For the binary patch-level classification models, the top five tiles associated with a predefined class chosen between the two are Base64 encoded and added as additional OBX segments (Additional file 1: Fig. S2B). For all slide-level classification models, in addition to the first OBX segment, two OBX segments containing the slide-level predicted label (or risk class) and the associated prediction score (or risk score) are added (Additional file 1: Fig. S2C).

After receiving the OUL^R21 message from the AI-DSS, the AP-LIS sends back an ACK message, following which the connection between the two is closed and the next message in the queue, if available, is processed. In the event of errors or failures, a maximum of three analysis attempts are programmed for the same slide. If none of the three attempts is successful, the next WSI in the queue, if available, is processed.

Communication between the AP-LIS and the AI-DSS was set-up via intranet connection using socket programming in a Python-based server-client architecture. The server-client architecture was developed to enable both server and client functionalities depending on the role of the AI-DSS at any given time point. In server mode, the AI-DSS listens for incoming OML^O33 messages and, upon receiving a connection, sends an ACK message to the AP-LIS, reads the OML^O33 message, and processes it. In client mode, the AI-DSS transmits DL inference results to the AP-LIS as OUL^R21 messages and remains listening for the ACK message, upon receipt of which the connection between the AP-LIS and the AI-DSS is closed. HL7 messaging exchange between the AP-LIS and the AI-DSS takes place via a Transmission Control Protocol/Internet Protocol (TCP/IP) connection using the Minimal Lower Layer Protocol (MLLP). The Python package Hl7apy v1.3.5 [36] was used to analyze and generate HL7 messages.

The datetime Python module was used to implement two counters that were run for six working days to automatically monitor the processing time of different components of the developed framework. In particular, the first counter tracked the total time required to process an analysis request (steps 1–7, Fig. 1). It was set off at the time the analysis request arrives to the AI-DSS via HL7 message and stopped at the time the final HL7 acknowledgment message sent by the AP-LIS is received by the AI-DSS. The second counter tracked the timing required to generate DL model inference results (steps 4 and 5, Fig. 1). It was set off at the beginning of DL model inference and stopped after the creation of the QuPath project.

### User study design

Nine months after the implementation of the integration framework, a survey was conducted in the pathology department of Gravina Hospital. The aim of the survey was to evaluate the user-friendliness and potential effectiveness of the developed framework in routine settings. To this aim, a questionnaire was sent electronically to a total of *N* = 5 pathologists. The five participants involved in the survey were attending pathologists with an average of 11.6 years of experience as pathologist (range: 5–25 years), including residency (Additional file 2: Sect. 1), with four of them with at least five years of experience in DP. Participants were asked to answer nine multiple-choice questions assessing their perception of the framework, and two optional free-text questions to collect additional comments (Additional file 2: Sects. 2/3). Results from the nine multiple-choice questions were summarized as barplots in R v4.2.2 [37] relying on the ggplot2 package v3.4.3 [38].

## Results

### The HL7-based framework enables a seamless integration of DL models in routine diagnostics

Our integration framework to incorporate DL models into the AP-LIS [39] was tested in the fully digitized pathology department of Caltagirone, Italy [17]. Relying on a Python-based server-client architecture, the framework was designed around two interconnected core elements: the AP-LIS and an external AI-DSS, encompassing the set of integrated DL models. Communication between the AP-LIS and the AI-DSS occurs through HL7 messaging, and free, open-source DP resources are used both for running DL model deployment on routine slides and visualizing DL model inference results.

Inference with patch-level classification models was performed relying on WSInfer [10]. Instead, inference with slide-level classification models relied on WSInfer-MIL [26] or marugoto [31]. Currently (last update June 2024), a total of 16 DL models can be applied to routine digitized H&E slides of the Caltagirone pathology department (Table 1). Eleven out of 16 DL models (*N* = 8 WSInfer built-in models; *N* = 3 in-house developed models) perform either a binary or a multi-class classification task to discriminate between different tissue types (e.g., epithelium, stroma, tumor, necrosis, and dysplasia) or conditions (e.g., tumor, non-tumor). The remaining five are publicly available models to predict the status of clinical biomarkers (i.e., *TP53*, *BRAF*, MSI) as well as the risk of cancer death in kidney renal papillary cell carcinoma (KIRP) and glioblastoma-lower grade glioma (GMBLGG).

**Table 1:** Pre-trained deep-learning models included in the integration framework

Model name	Reference	Model description	Classes	Visualization	Toolbox
**Patch-level classification models**					
**WSInfer built-in deep-learning models**					
pancancer-lymphocytes-inceptionv4.tcga	[40–42]	Lymphocyte detection	1. lymphocyte-negative 2. lymphocyte-positive	Measurement map	WSInfer
breast-tumor-resnet34.tcga-brca	[43–45]	Breast adenocarcinoma detection	1. no-tumor 2. tumor	Measurement map	WSInfer
lung-tumor-resnet34.tcga-luad	[46, 47]	Lung adenocarcinoma detection	1. lepidic 2. benign 3. acinar 4. micropapillary 5. mucinous 6. solid	Color map	WSInfer
pancreas-tumor-preactresnet34.tcga-paad	[48, 49]	Pancreatic adenocarcinoma detection	tumor-positive	Measurement map	WSInfer
prostate-tumor-resnet34.tcga-prad	[50, 51]	Prostate adenocarcinoma detection	1. grade3 2. grade4or5 3. benign	Color map	WSInfer
lymphnodes-tiatoolbox-resnet50.patchcamelyon	[52–56]	Lymph node metastasis detection in breast cancer	1. nomets 2. mets	Measurement map	WSInfer
colorectal-tiatoolbox-resnet50.kather100k	[53, 57]	Colorectal tissue classification	1. background 2. normal_colon_mucosa 3. debris 4. colorectal_adenocarcinoma_epithelium 5. adipose 6. mucus 7. smooth_muscle 8. cancer_associated_stroma 9. lymphocytes	Color map	WSInfer
colorectal-resnet34.penn	[58]	Colorectal tissue classification	1. epithelium 2. stroma 3. tumor 4. necrosis 5. dysplasia	Color map	WSInfer
**Custom developed deep-learning models**					
prostate-resnet18-caputo	(Caputo A, unpublished)	Prostate tumor detection	1. negative 2. positive	Measurement map	WSInfer
hpv-resnet50-merolla	(Merolla F, unpublished)	HPV detection	1. HPV negative 2. HPV positive	Measurement map	WSInfer
**Slide-level classification models**					
**WSInfer-MIL built-in deep-learning models**					
pancancer-tp53-mut.tcga	[27]	*TP53* mutation prediction	1. wildtype 2. mutant	Density map	WSInfer-MIL
gmblgg-survival-porpoise.tcga	[28, 29]	Survival prediction in glioblastoma-lower grade glioma	1. logits-time0 2. logits-time1 3. logits-time2 4. logits-time3	Density map	WSInfer-MIL
kirp-survival-porpoise.tcga	[28, 30]	Survival prediction in kidney renal papillary cell carcinoma	1. logits-time0 2. logits-time1 3. logits-time2 4. logits-time3	Density map	WSInfer-MIL
**Publicly available deep-learning models**					
braf-attMIL-marugoto	[32, 33]	Prediction of *BRAF* mutation in colorectal cancer	1. MUT 2. WT	Density map	marugoto
msi-attMIL-marugoto	[32, 33]	Prediction of MSI status in colorectal cancer	1. MSIH 2. nonMSIH	Density map	marugoto
**Custom developed deep-learning models**					
colorectal-mil-angeloni	(Angeloni M, unpublished)	Colorectal tissue classification	1. low-grade dysplasia 2. high-grade dysplasia 3. adenocarcinoma 4. other	Density map	WSInfer

For each deep-learning model, the table shows the following: a reference to the relevant publication(s) and/or model’s repository (column “Reference”), a summary description of the task performed (column “Model Description”), the classes provided in output (column “Classes”), the type of visualization heatmap chosen (column “Visualization”), and the toolbox used for model deployment (column “Toolbox”)

*GBM-LGG *Glioblastoma-lower grade glioma, *HPV *Human papillomavirus, *KIRP *Kidney renal papillary cell carcinoma, *met *Metastasis, *MSIH *Microsatellite instability high, *MUT *Mutated, *nomets *Non-metastasis, *WT *Wild type

**Fig. 2:**
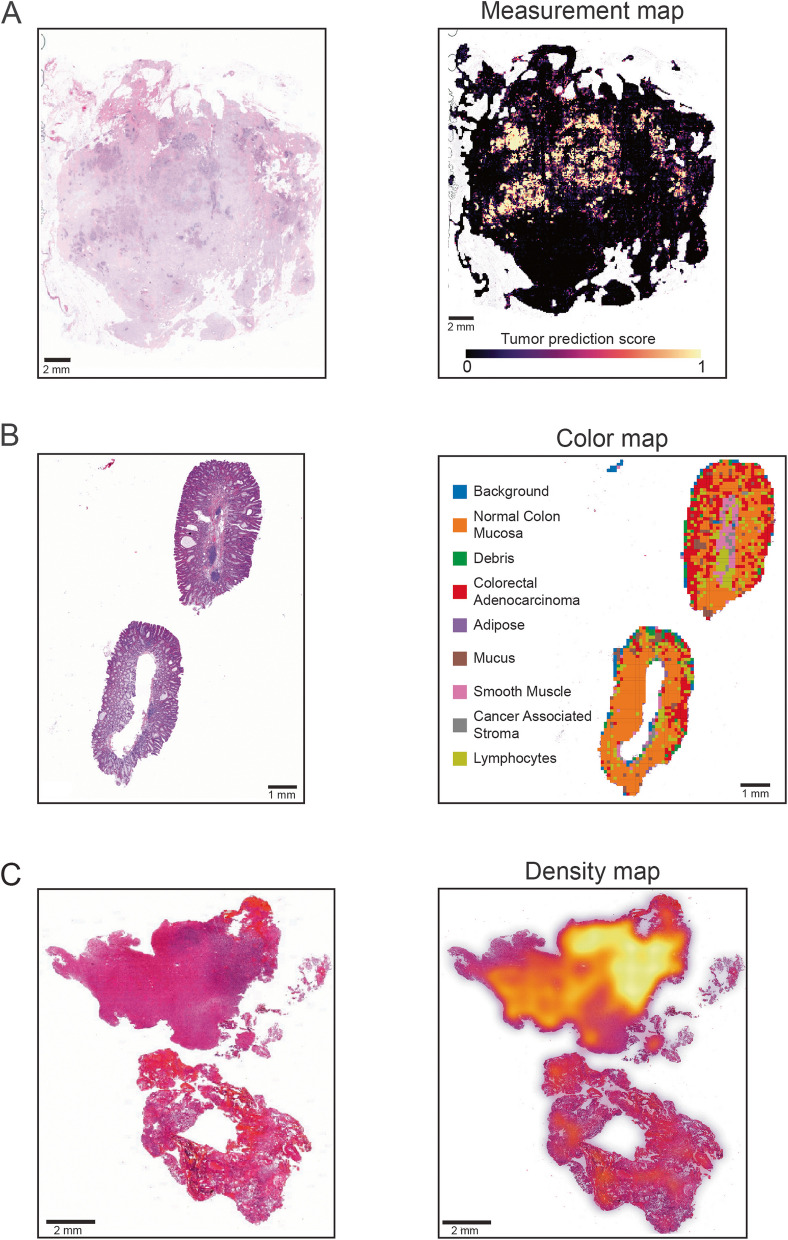
Visualization styles of deep-learning models output in QuPath. For each exemplary visualization style, the original H&E-stained image (left) and the corresponding colored heatmap (right) are shown. **A** Measurement map for binary classification obtained by applying the “breast-tumor-resnet34.tcga-brca” model on breast tissue. The measurement associated with a given tile refers to its prediction score for the class “Tumor”: the brighter the color, the higher the prediction score (based on the chosen color scale). **B** Color map for multi-class classification obtained by applying the “colorectal-tiatoolbox-resnet50.kather100k” model on a colon biopsy. Each tile is assigned to the class with the highest prediction score and colored accordingly. **C** Density map obtained by applying the “gmblgg-survival-porpoise.tcga” model on a glioblastoma resection. Here, brighter areas correspond to tiles associated with higher percentile-ranked attention scores. H&E hematoxylin and eosin

To facilitate pathologists in the interpretation of DL model predictions, an intuitive visualization of results was also implemented in our framework. To this aim, QuPath [18] and its pythonic interface paquo [35] were employed to create, for each analyzed WSI, a QuPath project with DL model inference results displayed as colored heatmap overlaid to the original WSI. Notably, based on the performed classification task, and whether or not an attention-based MIL approach was employed, three types of colored heatmaps were implemented in paquo: measurement maps, color maps, and density maps (Fig. 2). After analysis completion, the QuPath project can be conveniently opened directly from the virtual slide tray of the AP-LIS by clicking on the slide. Irrespective of the visualization style, when opening the QuPath project associated with a given WSI, a transparent overlay is already superimposed on the original slide and the heatmap visualization can be enabled from the QuPath graphical interface through few manual steps (for additional movie files see Additional file 3, Additional file 4, Additional file 5). Measurement maps were used to visualize the output of binary classification tasks (e.g., tumor, non-tumor). In QuPath, measurement maps provide a color-coded representation of a measure associated with a given detection object. Here, as measure for each tile detection object we used the prediction score associated with a predefined class between the two, such as the worst class from the clinical point of view (e.g., tumor) (Fig. 2A, Additional file 3). Multi-class classification problems (e.g., classification of epithelium, stroma, tumor, necrosis, and dysplasia in colorectal samples) were visualized through color maps. Here, the QuPath project was built to support a number of pre-defined annotation classes, each assigned a distinct color, matching the number of labels predicted by the multi-class DL model. Tiles within the tissue sample are colored according to the color of the annotation class corresponding to the predicted label (Fig. 2B, Additional file 4). As visualization style for attention-based MIL models, density maps were implemented. Attention-based MIL models provide in output an overall slide-level prediction score, with attention scores available at tile-level. Density maps in QuPath were originally conceived to help finding hotspots for specific clinical applications (e.g., Ki67 scoring), and more in general to find areas with high object density. Here, we relied on density maps to highlight tiles, and thus areas, associated with higher attention scores (Fig. 2C, Additional file 5). As additional support to pathologists in interpreting the output of MIL models, a summary of the predicted label (or risk class) and associated prediction score (or risk score) were also provided as description of the QuPath project.

**Fig. 3:**
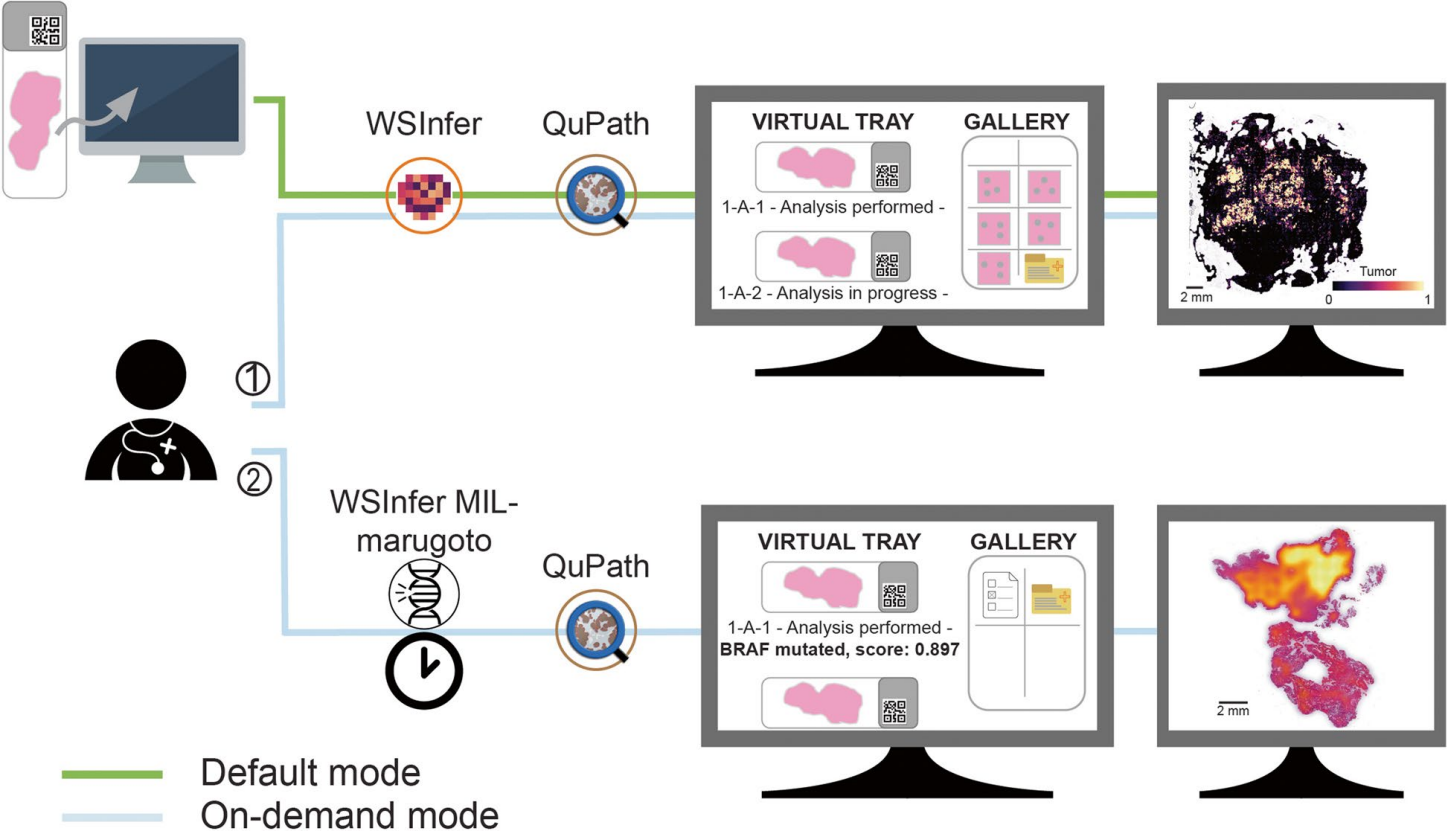
Operational modalities of the integration framework. In the default mode (green path) new digitized slides are automatically analyzed using the WSInfer toolbox for deployment of both built-in and custom developed patch-level classification models. In this operational modality, a colored heatmap is created for each analyzed slide and QuPath is used to visualize deep-learning (DL) model inference results. For a selected subset of models, the gallery is populated with the top five predicted tiles supporting the prediction. In the on-demand mode (light blue path), an analysis request is manually initiated by pathologists directly from the virtual slide tray. This request allows the deployment (1) of the same models available in the default mode and (2) of additional DL models for prediction of clinical biomarkers and risk of cancer-related death through the WSInfer-MIL and the marugoto toolboxes. In the latter case, slide-level predictions are displayed as slide description in the virtual slide tray and the visualization of model predictions in QuPath through density maps is also provided

**Fig. 4:**
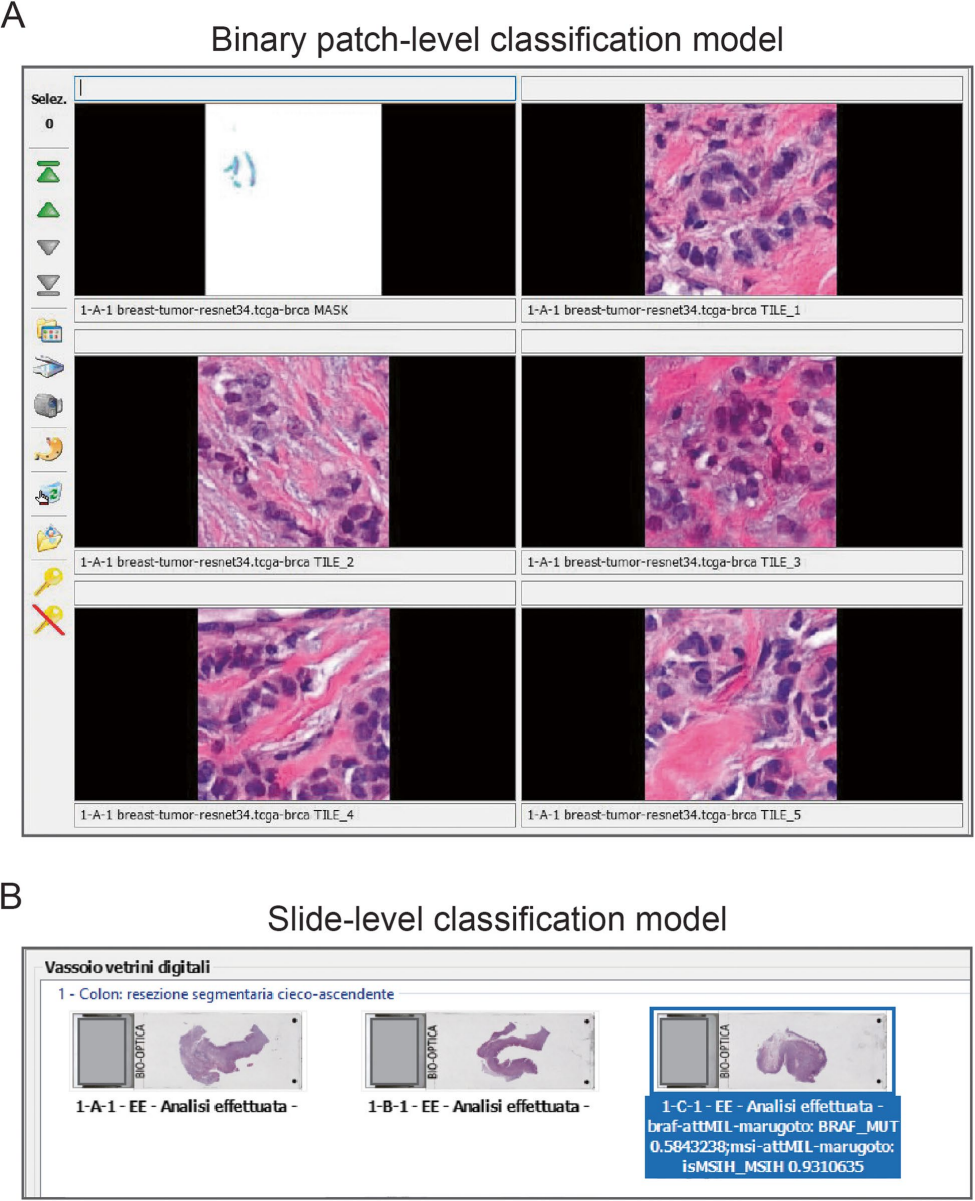
Integration of deep-learning model predictions in the virtual slide tray. **A** Example of binary patch-level classification model results integrated in the gallery of the anatomic pathology laboratory information system (AP-LIS) showing on the top-left the tissue mask used for patches generation and after it the top five predicted tiles for the class “Tumor.” **B** Example of slide-level classification model results visualized in the virtual slide tray of the AP-LIS as slide description. The whole-slide image (WSI) marked by the blue box was analyzed using the marugoto toolbox with both the “braf-attMIL-marugoto” and the “msi-attMIL-marugoto” models and predicted as *BRAF* mutated with a prediction score of 0.58 and microsatellite unstable with a prediction score of 0.93. EE Ematossilina–Eosina [Hematoxylin&Eosin]; Analisi effettuata [Analysis performed]

The developed integration framework is blind to the specific scanner proprietary image format, as long as the input HL7 message provides the path to the WSI to analyze. At present the framework has been set up to process two different image formats (.mrxs and.svs) and could be easily adapted to other scanners. Furthermore, it does not require any user manual intervention for the deployment of DL models on routine slides. Indeed, the interaction between the AP-LIS and the AI-DSS initiates automatically in conjunction with an OML^O33 request transmitted from the AP-LIS to the AI-DSS. The “default” deployment mode runs continuously as each new slide is digitized, automatically selecting one or more DL models to apply on the basis of tissue type and staining (Fig. 3). Upon digitization completion, the digitized slide becomes readily available in the AP-LIS, then it is stored in the queue of the slides to analyze and processed according to the FIFO principle. With the “on-demand” deployment mode, DL model inference is initiated by pathologists by choosing the model to apply directly from a drop-down menu on the virtual slide tray (Fig. 3, Additional file 1: Fig. S3). Analogously to the default deployment mode, also in the on-demand mode the slide is stored in the queue as soon as the pathologist selects the DL model to apply and then processed according to the FIFO principle. The on-demand mode is particularly useful to (i) re-analyze cases assigned at the acceptance stage to a wrong tissue type, and therefore analyzed by default with a wrong DL model; (ii) re-analyze WSIs whose analysis failed in default-mode; (iii) run DL models for the prediction of clinical biomarkers or cancer-related risk death after a confirmed diagnosis of cancer. WSIs analysis status, including potential failures, can be monitored from the AP-LIS through the virtual slide tray (Additional file 1: Fig. S4A). Upon analysis completion, DL model predictions are transmitted from the AI-DSS to the AP-LIS as OBX segments via OUL^R21 message. For all models results can be visualized in QuPath. In addition, for binary patch-level classification models, the top five tiles with the highest prediction probability associated with the predefined class are included in the gallery as JPEG files (Fig. 4A). Instead, for slide-level classification models, the predicted class (or risk class) and the associated prediction score (or risk score) are displayed as WSI description in the virtual slide tray (Fig. 4B). If multiple DL models are run on the same WSI, opening the slide in the virtual slide tray will display a pop-up window listing all deployed model names, and pathologists can choose which model results to visualize in QuPath through a double click (Additional file 1: Fig. S4B).

The time taken by the developed framework to process analysis requests was monitored for six working days, for a total of 452 analysis requests. The median time taken for the complete analysis of a given WSI (steps 1–7 in Fig. 1) was 2.47 min (range, 29 s–17.58 min). In particular, the median time taken to run DL model deployment and create the QuPath project to visualize model inference results (steps 4 and 5 in Fig. 1) was 18 s (range, 8 s–4.7 min).

Taken together, our framework allows a seamless, user-friendly integration of several DL models in the AP-LIS, thus making them directly accessible by pathologists during their routine diagnostics workflow. Furthermore, the intuitive visualization styles implemented allow pathologists to explore and get insights into DL model predictions, thus helping in overcoming the black-box nature of the adopted CPath solution.

### User study highlights the user-friendliness and potential effectiveness of the integration framework

To assess the suitability of the framework for implementation in clinical care, its user-friendliness and potential effectiveness were assessed by five pathologists through a user study questionnaire (Additional file 2). When evaluating the user-friendliness of the framework (Additional file 1: Fig. S5A), all responders found the overall framework very intuitive or intuitive. Participants unanimously reported a very intuitive, almost immediate visualization of DL model inference results in the virtual slide tray. Regarding the usage of the QuPath software component of the framework, four participants found the visualization in QuPath intuitive or very intuitive, and all participants reported a very fast or adequate speed of visualization of DL model inference results as colored heatmaps. When evaluating the on-demand mode, four participants indicated that the launch of on-demand requests was very intuitive, almost immediate and all participants reported an adequate speed of running. In additional free-text comments, two participants remarked the overall ease of use of the workflow, the minimal training required, and the benefits of visualizing DL model results directly in the virtual slide tray. When evaluating the potential effectiveness of the framework (Additional file 1: Fig. S5B), four responders answered that it could be very helpful in the clinical practice and one that it could be somewhat helpful. More specifically, the framework was perceived as either very or somewhat helpful in reducing diagnostic-related times and/or costs. Ultimately, three participants found that the framework could be very helpful for patients.

Taken together, these insights indicate that the developed integration framework is well-received by practicing pathologists, suggesting its strong usability and potential effectiveness in clinical practice.

## Discussion

The potential of AI tools in pathology to enhance diagnosis, prognosis and prediction of clinical biomarkers has been widely acknowledged, marking the onset of the “third revolution” in pathology [59]. However, the difficulty to achieve a full integration of these tools into clinical settings is one of the key factors contributing to their scarce adoption in routine diagnostics. Here, we leveraged a fully digitized pathology infrastructure [17] to develop a standardized, open-source Python-based framework to integrate both publicly available and custom developed DL models into the AP-LIS, and visualize and interpret DL model results through colored heatmaps. Importantly, the integration was based on HL7, one of most relevant electronic health record standards to exchange electronic healthcare data between medical information systems [60]. HL7 was chosen as integration standard since it had been already used in Gravina Hospital’s pathology department to interconnect the AP-LIS with other laboratory instrumentation including slide scanners and stainers. Furthermore, at present, in Italy the exchange, integration, and retrieval of health data still heavily rely on HL7. However, other healthcare integration standards such as the more recent fast healthcare interoperability resources (FHIR), could be used. Of note, irrespectively of whether HL7 or FHIR is adopted, the backbone of the whole integration framework, namely the interconnection between the AP-LIS and the AI-DSS, would remain unchanged. The only difference would involve the format of the sent and received messages, i.e., JSON files with FHIR and text-pipe-and-hats with HL7. Thus, given the ongoing transition towards FHIR, there is no obstacle to adapt the proposed integration framework to use this standard in the future. The employment of freely-available resources for both DL models deployment and visualization of model results, coupled with HL7 messaging, reduces implementation costs, and enhances the framework’s portability across different healthcare environments. Also, the data transfer speed in which the framework operates aligns with the capabilities of standard laboratory network infrastructures, thus further contributing to make our implementation feasible in most pathology departments.

The proposed integration strategy has been designed to be model-agnostic, without being tied to a particular DL model’s architecture. Indeed, as long as a pre-trained DL model is available (e.g., as.pt file or.pth file), this can be seamlessly included into our framework for deployment on new routine slides. Thus, also models obtained via fine-tuning of pathology foundation models [61–66] on domain-specific classification tasks as well as models derived using vision-language models [67–71] as image encoders followed by a linear probing protocol [69, 72, 73] can be readily incorporated. WSInfer [10] and WSInfer-MIL were chosen for the deployment of pre-trained DL models since they implement a streamlined, fast, end-to-end pipeline that goes from tissue detection to inference with minimal user intervention. Our framework successfully supported different types of classification tasks, from strongly supervised to attention-based MIL frameworks, from tumor detection, to prediction of prognostic clinical biomarkers (e.g., *TP53* mutation) and risk of cancer-related death. The integration of marugoto as additional toolbox for the prediction of *BRAF* mutational status and MSI, further highlighted the flexibility and adaptability of the framework towards publicly available resources, in addition to extending the pool of integrated models for the prediction of predictive clinical biomarkers.

The open-source bio-image analysis tool QuPath [18] was used to visualize DL model inference results. QuPath had been already fully integrated within the AP-LIS of the Caltagirone digital lab in April 2023 since it not only allows WSI visualization, but it also supports high-throughput, reliable image analyses, enabling objective quantification of clinically relevant biomarkers in different cancer entities [74]. One of the major limitations of commercial AI solutions is often the requirement for specific visualization software that does not easily integrate with existing AP-LIS or picture archiving and communication systems (PACS) [8], thus ultimately contributing to the slow uptake of AI technologies in the clinical practice. In contrast, QuPath provides a comprehensive visualization capability, irrespective of the type of DL model employed.

We implemented a “default” operational mode, whereby model deployment runs automatically as each new slide is digitized, and an “on-demand” operational mode whereby model inference is initiated by pathologists from the virtual slide tray. For instance, an on-demand request for *BRAF* mutational status can be initiated after the confirmation by pathologists of a cancer diagnosis provided by a previously applied classification model. In fact, in the current framework, the deployment of predictive and prognostic models is only available in the on-demand mode. Yet this design choice could be easily modified and the deployment of these models included also in the default mode. In the default mode, the use of a manually curated correspondence table between tissue type, staining, and DL models guarantees that an appropriate model is always deployed. The only step of the entire digital pathology workflow that can potentially lead to deploy an incorrect model is a wrong tissue type assignment at sample admission. However, this extremely rare event can be easily controlled and corrected throughout sample’s processing (e.g., during grossing), or through an on-demand analysis request, where the choice of the DL model to deploy is left to the user. These two developed operational modalities have been previously referred to as “preprocessing fashion” and “on-demand fashion” [75]. Furthermore, they match what has been advocated in a recent review, which highlighted how the efficiency of AI tools will be fully exploited only when the integrated AI solution can run automatically on the backend or be specifically triggered at need during diagnostics [8].

Collectively, our study shows that implementing CPath solutions into a fully digitized pathology department is feasible and can be optimized to be highly user-friendly for pathologists. This was confirmed by our user study, whose primary goal was to assess the effectiveness and user-friendliness of the proposed integration framework, which are critical aspects when evaluating practical integration in routine settings. Pathologists additionally found the framework potentially very helpful in clinical practice. Indeed, the possible applications of such workflow are extensive. For example, a foreseeable scenario might be one in which a DL model for MSI detection in colorectal cancer serves as AI-based pre-screening tool to rule-out non-MSI cases [76]. In light of the costs, especially when using larger next generation sequencing panels, such a pre-screening tool could help in reducing the financial impact of MSI testing. In addition, it would allow to speed up turnaround times for clinical decision making by prioritizing cases. The establishment of our framework will facilitate future studies aimed at assessing the patients’ and healthcare benefits from the integration of DL models in routine diagnostics.

Our framework, though, despite its strengths and novelty, faces limitations. All software (except for the AP-LIS) and DL models employed are non-commercial, open-source resources intended for research use only. Hence, the use of the integration framework outside of research context is under the responsibility of the user. Indeed, in order for a CPath solution to be deployed for clinical use, rigorous validation and regulatory approval (e.g., FDA or IVD) are crucial requirements. These requirements are even more important when considering ethical and liability concerns raising from the application of AI tools in diagnostics [77–82]. In the present study none of the results of the integrated DL models were used to make clinical decisions, nor were they clinically validated. The choice of the models included in the integration framework was functional to test the overall workflow. Namely, available pre-trained DL models covering a diverse range of classification tasks and tissue types were selected. With regard to clinical validation, each laboratory should make decisions depending on its specific diagnostics needs and instruments, but especially on the rapidly evolving regulations [80, 81]. The use of models trained using data from multiple institutions and representative of the target population should be favored [83], as these models have been shown to generalize quite well to data from other institutions [84]. The question of how DL-based assessments can be included in diagnostic reports is also still open. Recommendations should be made for the diverse potential use of AI-based algorithms, e.g., as support for the diagnosis or as independent validated diagnostic tools. Very recently, the European Society of Radiology has published recommendations for the use of AI in radiology [85]. It would be desirable for governments and the scientific communities to work together to formulate clear recommendations also in the field of pathology.

In order to use our framework, digitization of the pathology department is the major requirement and the primary financial investment. Also, an intense and close collaboration between clinicians, technicians, and IT services is crucial. It is worth nothing, though, that the benefits of this investment could extend beyond the pathology workflow to involve the whole hospital infrastructure. Furthermore, once the core DP infrastructure is in place (e.g., scanners, storage, AP-LIS), thanks to the freely available resources utilized by our implementation, the set-up of the integration framework would need only minimal investments.

Ultimately, pathologists’ acceptance and proficiency in utilizing AI tools represent other barriers. The need for integrating computational skills into pathology residency programs emerged as early as 2016 [86]. As it is becoming more and more crucial for clinicians to be able to interpret the output of AI technologies, the inclusion of digital health competencies in physicians’ ongoing training is of paramount importance.

## Conclusions

Our proposed framework provides a standardized, portable, and open-source solution able to run DL models on routine slides relying on HL7 messaging and freely available DP software. This solution allows, for the first time, to effectively close the gap between research on AI-based tools and their clinical implementation, thus marking a first step towards their advocated “realistic assessment” [87] in routine settings.

## Supplementary Information

Additional file 1: Fig. S1. Example of OML^O33 HL7 message; Fig. S2. Examples of OUL^R21 HL7 messages; Fig. S3. Snapshot of the virtual slide tray of the AP-LIS during an on-demand analysis request; Fig. S4. Snapshot of the virtual slide tray of the AP-LIS following an OML^O33 request; Fig. S5. Results of the user study questionnaire.

Additional file 2. User study questionnaire administered to pathologists. Content of the user study questionnaire administered to pathologists to evaluate the integration framework in terms of user-friendliness and potential effectiveness. Mandatory fields are marked with an asterisk (*).

Additional file 3. Activation of measurement map visualization in QuPath. Measurement map visualization is enabled by (1) navigating to Measure > Show measurement map (or alternatively pressing simultaneously Ctrl+Shift+M), (2) selecting the label measurement (e.g., Tumor) popping up in the Measurement map window, (3) toggling on the detection objects through the “Show/hide detection objects” toolbar button, and (4) filling up the detection objects through the “Fill/Unfill detection object ROIs for display” toolbar button.

Additional file 4. Activation of color map visualization in QuPath. Color map visualization is enabled by filling up the detection objects through the “Fill/Unfill detection object ROIs for display” toolbar button. The predicted classes can be visualized by navigating to the “Annotations" tab on the left.

Additional file 5. Activation of density map visualization in QuPath. Density map visualization is enabled by (1) navigating to Analyze > Density maps > Create density map. (2) Density map appearance can be modified by changing the density type to, e.g., Gaussian-weighted. Slide-level outcome is provided as free text in the white box on the bottom left panel under the Project tab.

## Data Availability

The patient slides used in this study cannot be made publicly available due to general data protection regulations and institutional guidelines.

## Abbreviations

AI: Artificial intelligence
AI-DSS: Artificial intelligence-based decision support system
AP-LIS: Anatomic pathology laboratory information system
CLI: Command-line
CPath: Computational pathology
DP: Digital pathology
DL: Deep-learning
FDA: Food and drug administration
FHIR: Fast healthcare interoperability resources
FIFO: First in first out
GMBLGG: Glioblastoma-lower grade glioma
GPU: Graphics processing unit
HL7: Health level 7
H&E: Hematoxylin and eosin
IT: Information technology
IVD: In vitro diagnostics
KIRP: Kidney renal papillary cell carcinoma
LTS: Long-term support
MIL: Multiple-instance learning
MLLP: Minimal lower layer protocol
mpp: Microns per pixel
MSI: Microsatellite instability
NAS: Network-attached storage
PACS: Picture archiving and communication systems
TCP/IP: Transmission control protocol/internet protocol
WSI: Whole-slide image
