# Supplementary material for: Closing the gap in the clinical adoption of computational pathology: a standardized, open-source framework to integrate deep-learning models into the laboratory information system
Source: Genome Med. 2025 May 26;17:60. doi: 10.1186/s13073-025-01484-y (PMC12107920; doi:10.1186/s13073-025-01484-y)
Supplement: Supplementary file 1 — Additional file 1: Fig. S1. Example of OML^O33 HL7 message; Fig. S2. Examples of OUL^R21 HL7 messages; Fig. S3. Snapshot of the virtual slide tray of the AP-LIS during an on-demand analysis request; Fig. S4. Snapshot of the virtual slide tray of the AP-LIS following an OML^O33 request; Fig. S5. Results of the user study questionnaire. [file 13073_2025_1484_MOESM1_ESM.pdf]

Closing the gap in the clinical adoption of computational pathology: a standardized, open-source framework to integrate deep-learning models into the laboratory information system

Angeloni M *et al.*

Additional file 1

Contents

Fig. S1 - page 2
Fig. S2 - page 3
Fig. S3 - page 4
Fig. S4 - page 5
Fig. S5 - page 6

```
MSH|^~\&|ZZZ|WWW|XXX|YYYY|20240322080152||OML^O33^OML_O33|2857198947|P|2.6

PID|||PRVPRV43S04A075M^^^^CF~153042^^^^MPI~207444994^^^^CS||NAME^SURNAME||194
31104|F|||ADDRESS|||||||||||||

SPM|1|18584052||MODEL_NAME^MODEL_NAME|||||||||notes|||20240322100000|

ORC|NW|SLIDEBARCODE|| 18-B-01806|SC||||20240322100000|||MDMMDM63M29G224V
^CLINICIAN^CLINICIAN|

OBR|1|SLIDEBARCODE||HE^HE||20240322100000||||||/PATH/TO/ARCHIVE/SLIDE_ID
```

Fig. S1. Example of OML^O33 HL7 message. Example of laboratory order (OML^O33) HL7 version 2.6 message transmitted from the anatomic pathology laboratory information system (AP-LIS) to the artificial intelligence-based decision support system (AI-DSS) for the analysis of a whole-slide image (WSI). Each OML^O33 message is made up of five segments, i.e., MSH, PID, SPM, ORC, and OBR (bold black). The name of the deep-learning model to deploy is stored in the fields 4.1 and 4.2 of the SPM segment (bold orange), whereas the path to the WSI to analyze is retrieved from field 13 of the OBR segment (bold green).

A

MSH|^~\&|XXX|YYY|ZZZ|WWW|20240322090848||OUL^R21|0001928917|P|2.6

PID|||PRVPRV43S04A075M^^^^CF~153042^^^^MPI~207444994^^^^CS||NAME^SURNAME||194 31104|F|||ADDRESS||||||||||||||

ORC|NW|SLIDEBARCODE|| 18-B-01806|SC||||20240322100000|||MDMMDM63M29G224V ^CLINICIAN^CLINICIAN|

OBR|1|SLIDEBARCODE||HE^HE||20240322100000|||||||/PATH/TO/ARCHIVE/SLIDE_ID

OBX|1|ST|SLIDEBARCODE^MODEL||MODEL_NAME||||||F

OBX|2|ED|SLIDEBARCODE^RUN||b‘run_metadata.json’||||||F

OBX|3|ED|SLIDEBARCODE^MASK||b‘mask.jpg’||||||F

OBX|4|ED|SLIDEBARCODE^TABLE||b‘models_output.csv’||||||F

B

MSH|^~\&|XXX|YYY|ZZZ|WWW|20240322090848||OUL^R21|0001928917|P|2.6

PID|||PRVPRV43S04A075M^^^^CF~153042^^^^MPI~207444994^^^^CS||NAME^SURNAME||194 31104|F|||ADDRESS||||||||||||||

ORC|NW|SLIDEBARCODE|| 18-B-01806|SC||||20240322100000|||MDMMDM63M29G224V ^CLINICIAN^CLINICIAN|

OBR|1|SLIDEBARCODE||HE^HE||20240322100000|||||||/PATH/TO/ARCHIVE/SLIDE_ID

OBX|1|ST|SLIDEBARCODE^MODEL||MODEL_NAME||||||F

OBX|2|ED|SLIDEBARCODE^RUN||b‘run_metadata.json’||||||F

OBX|3|ED|SLIDEBARCODE^MASK||b‘mask.jpg’||||||F

OBX|4|ED|SLIDEBARCODE^TABLE||b‘models_output.csv’||||||F

OBX|5|ED|SLIDEBARCODE^TILE||b‘tile1.jpg’||||||F

OBX|6|ED|SLIDEBARCODE^TILE||b‘tile2.jpg’||||||F

OBX|7|ED|SLIDEBARCODE^TILE||b‘tile3.jpg’||||||F

OBX|8|ED|SLIDEBARCODE^TILE||b‘tile4.jpg’||||||F

OBX|9|ED|SLIDEBARCODE^TILE||b‘tile5.jpg’||||||F

C

MSH|^~\&|XXX|YYY|ZZZ|WWW|20240322090848||OUL^R21|0001928917|P|2.6

PID|||PRVPRV43S04A075M^^^^CF~153042^^^^MPI~207444994^^^^CS||NAME^SURNAME||194 31104|F|||ADDRESS||||||||||||||

ORC|NW|SLIDEBARCODE|| 18-B-01806|SC||||20240322100000|||MDMMDM63M29G224V ^CLINICIAN^CLINICIAN|

OBR|1|SLIDEBARCODE||HE^HE||20240322100000|||||||/PATH/TO/ARCHIVE/SLIDE_ID

OBX|1|ST|SLIDEBARCODE^MODEL||MODEL_NAME||||||F

OBX|2|ST|SLIDEBARCODE^PRED_LABEL||TP53_MUT||||||F

OBX|3|NM|SLIDEBARCODE^PRED_SCORE||0.87||||||F

Fig. S2. Examples of OUL^R21 HL7 messages. Examples of unsolicited laboratory observation (OUL^R21) HL7 version 2.6 messages transmitted from the artificial intelligence-based decision support system (AI-DSS) to the anatomic pathology laboratory information system (AP-LIS) : **A** after running model deployment with pre-trained patch-level classification models, **B** same as **A** but with the addition of the top five predicted tiles, and **C** after running model deployment with pre-trained slide-level classification models.

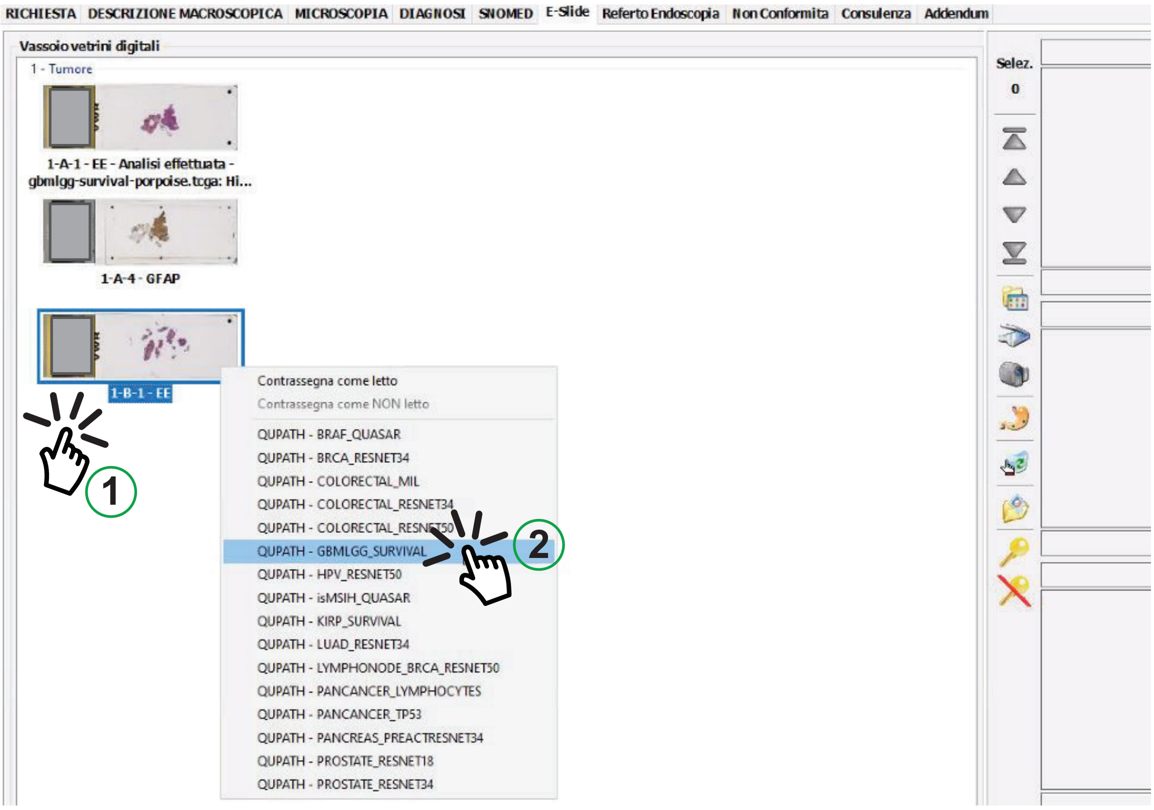

Fig. S3. Snapshot of the virtual slide tray of the AP-LIS during an on-demand analysis request. Pathologists can initiate an analysis request directly from the virtual slide tray by first right-clicking on the whole-slide image to analyze, and then double clicking on the deep-learning model (DL) of choice among the list of integrated DL models. AP-LIS = anatomic pathology laboratory information system.

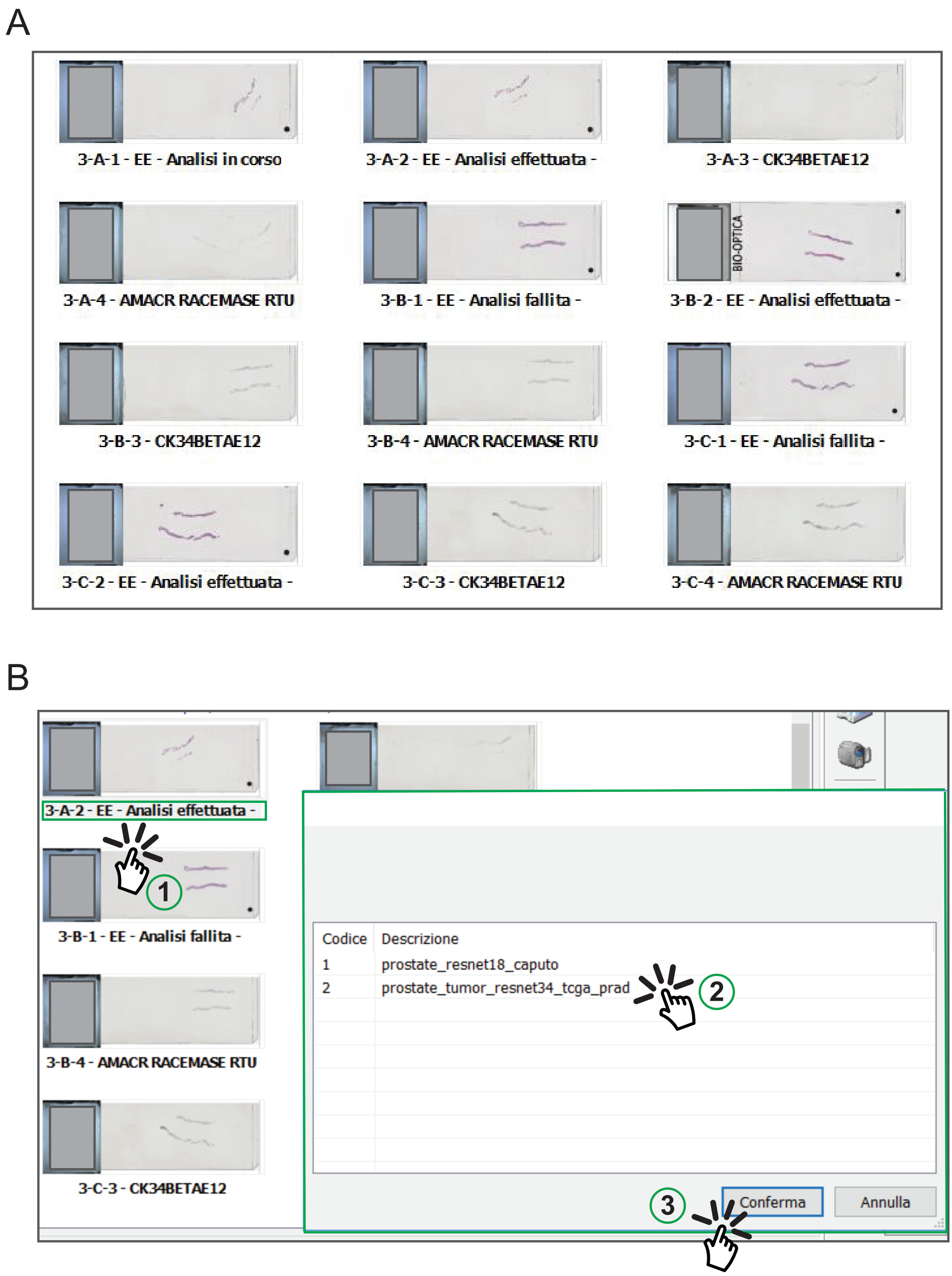

Fig. S4. Snapshot of the virtual slide tray of the AP-LIS following an OML^O33 request. **A** Overview of the virtual slide tray of the anatomic pathology laboratory information system (AP-LIS) with multiple whole-slide images (WSIs) for the same patient. WSIs flagged as "Analisi in corso" [Analysis in progress] (i.e., 3-A-1) are those for which an analysis request has been sent from the AP-LIS to the artificial intelligence-based decision support system (AI-DSS), and are currently in a queue waiting for deep-learning (DL) model deployment. WSIs successfully analyzed are flagged as "Analisi effettuata" [Analysis performed] (e.g., 3-A-2). WSIs whose analysis resulted in an error are flagged as "Analisi fallita" [Analysis failed] (e.g., 3-B-1). **B** Pop-up window listing all DL models already run on a WSI and for which results can be visualized as colored heatmap in QuPath. EE -Ematossilina-Eosina [Hematoxylin&Eosin]; Codice [Code]; Descrizione [Description]; Conferma [Confirm]; Annulla [Cancel].

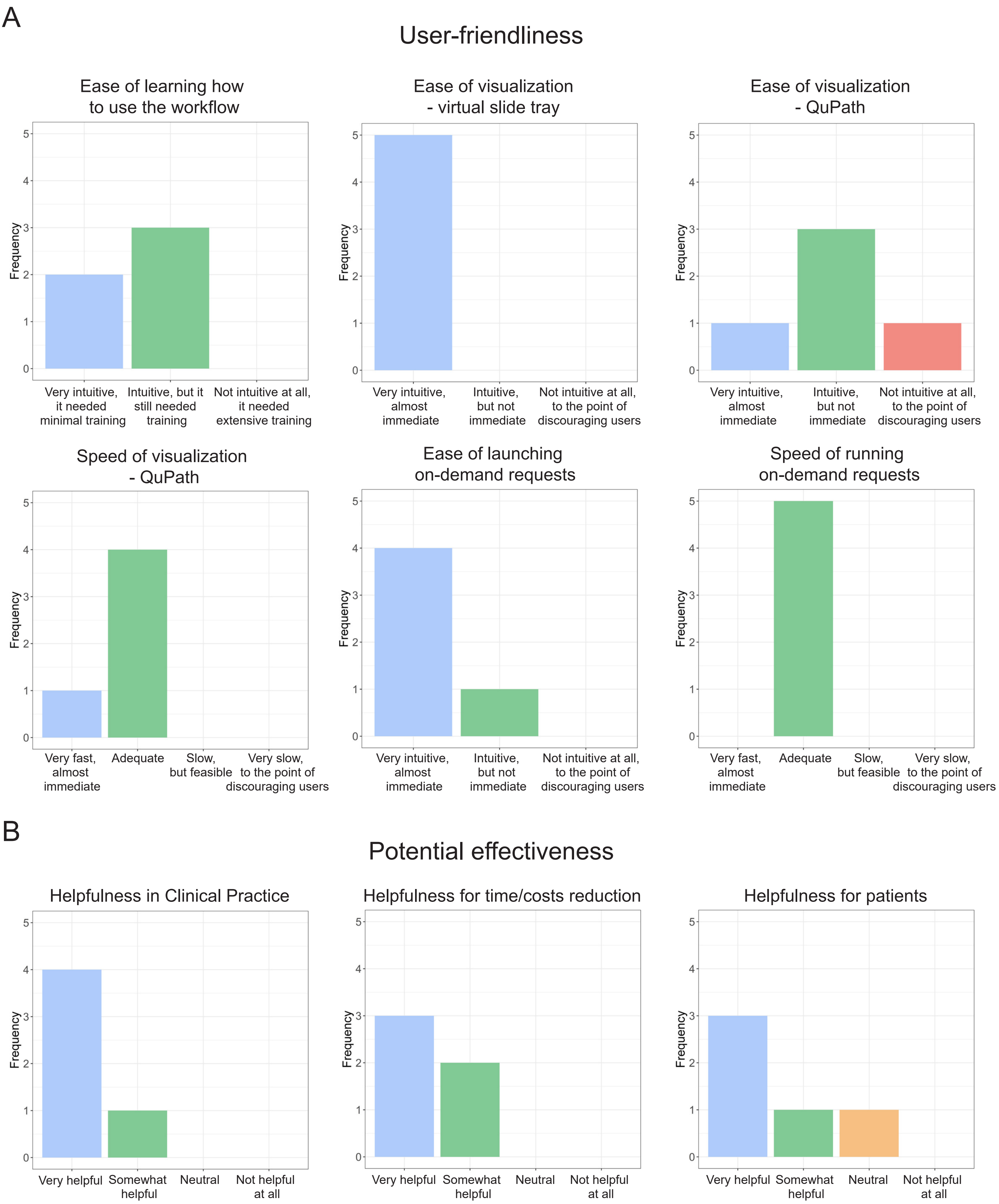

Fig. S5. Results of the user study questionnaire. For each multiple-choice question aimed at assessing **A** the user friendliness and **B** the potential effectiveness of the developed integration framework, a barplot distribution of participants' responses is provided.
