# Supplementary material for: Closing the gap in the clinical adoption of computational pathology: a standardized, open-source framework to integrate deep-learning models into the laboratory information system
Source: Genome Med. 2025 May 26;17:60. doi: 10.1186/s13073-025-01484-y (PMC12107920; doi:10.1186/s13073-025-01484-y)
Supplement: Supplementary file 2 — Additional file 2. User study questionnaire administered to pathologists. Content of the user study questionnaire administered to pathologists to evaluate the integration framework in terms of user-friendliness and potential effectiveness. Mandatory fields are marked with an asterisk (*). [file 13073_2025_1484_MOESM2_ESM.pdf]

Closing the gap in the clinical adoption of computational pathology: a standardized, open-source framework to integrate deep-learning models into the laboratory information system

Angeloni M *et al.*

Additional file 2

Contents

User study questionnaire administered to pathologists. Content of the user study questionnaire administered to pathologists to evaluate the integration framework in terms of user-friendliness and potential effectiveness. Mandatory fields are marked with an asterisk (*).

Integration Framework Evaluation Questionnaire

On the basis of your experience, please evaluate in terms of user-friendliness and potential effectiveness the framework to integrate deep-learning models in the routine diagnostic workflow of the Gravina Hospital pathology department.

Section 1

1) Please select your role*

- ☐ Attending pathologist
- ☐ Resident pathologist

2) Please indicate your years of experience as pathologist (including residency) *

____

Section 2

These questions will assess your perception of the user-friendliness of the system.

1) Ease of learning how to use the workflow*

How intuitive is the use of the workflow? Did you need extensive training to learn how to use it?

- ☐ Not intuitive at all, it needed extensive training
- ☐ Intuitive, but it still needed training
- ☐ Very intuitive, it needed minimal training

2) Ease of visualization - virtual slide tray *

Is the visualization in the virtual slide tray of the DL analysis status and DL model results intuitive?

- ☐ Not intuitive at all, to the point of discouraging users
- ☐ Intuitive, but not immediate
- ☐ Very intuitive, almost immediate

3) Ease of visualization – QuPath*

Is the visualization in QuPath of the DL model results (measurement maps, color maps, density maps...) intuitive?

- ☐ Not intuitive at all, to the point of discouraging users
- ☐ Intuitive, but not immediate
- ☐ Very intuitive, almost immediate

4) Speed of visualization - QuPath*

How fast is the opening in QuPath of DL model results?

- ☐ Very slow, to the point of discouraging users
- ☐ Slow, but feasible
- ☐ Adequate
- ☐ Very fast, almost immediate

5) Ease of launching on-demand requests*

How intuitive is to request an on-demand analysis for a slide?

- ☐ Not intuitive at all, to the point of discouraging users
- ☐ Intuitive, but not immediate
- ☐ Very intuitive, almost immediate

6) Speed of running on-demand requests*

How fast is the workflow in processing an on-demand request?

- ☐ Very slow, to the point of discouraging users
- ☐ Slow, but feasible
- ☐ Adequate
- ☐ Very fast, almost immediate

7) Do you have any further comments on the user-friendliness of the workflow?

____

____

____

Section 3

These questions will assess your perception of the potential effectiveness of the system.

1) Helpfulness in Clinical Practice*

Do you think the overall workflow could be helpful in the clinical practice?

- ☐ Not helpful at all
- ☐ Neutral
- ☐ Somewhat helpful
- ☐ Very helpful

2) Helpfulness for time/cost reduction*

Do you think the workflow could be helpful in reducing diagnostic-related time and/or costs?

- ☐ Not helpful at all
- ☐ Neutral
- ☐ Somewhat helpful
- ☐ Very helpful

3) Helpfulness for patients*

Do you think this workflow could be helpful in improving diagnosis and/or patient outcomes?

- ☐ Not helpful at all
- ☐ Neutral
- ☐ Somewhat helpful
- ☐ Very helpful

4) Do you have further comments on the potential effectiveness of the workflow?

____

____

____
